# Supplementary material for: Developmental alterations in centrosome integrity contribute to the post-mitotic state of mammalian cardiomyocytes
Source: eLife. 2015 Aug 6;4:e05563. doi: 10.7554/eLife.05563 (PMC4541494; doi:10.7554/eLife.05563)
Supplement: Supplementary file 1. — Homology analysis of PCNT to determine conserved regions for the identification of PCNT splice isoforms in the adult zebrafish heart. DOI: http://dx.doi.org/10.7554/eLife.05563.012 [file elife05563s001.docx]

mouse GCTATT-----------------------GTGATCCTCCTGTCTCTAACCCTTT-GGAGC

human GCATTC-----------------------ATGGTGTT---GTGCGTAGTTCAGT-GGTGC

rat GTTTTTTGACTGGAAGCCAACCAGTCCCTGTGATCCTCCTGCCTCTAACCCTCTCGGAGC

zebrafish GCAGTC-----------------------ACGGGAAC--TGTAGTTGATC--ATCAGCGC

* * * * * * * **

mouse TGG---GTTTGTGATGTGCTGTTCAGGACACCTGGGTTGCTACATGGATGCTGATTTCTG

human AGGCACATTCACGGTGT--TGT-----ACATTCGG--TG----ATGGAATCACATTC---

rat TGG---GTTTAGGACGTGTTGTTCAGGATGCCTGGGTTGCTATGTGGATGCTGATTTCTG

zebrafish AAT---ATTCATGAT----TGT----AAAA---GAACTAC--------ATCAACTTTTTA

** * *** * * * * **

mouse AGCTGCAGTCCTCATGGCTGCCCAAATGCTCTTAACCACCGAGCCATCTCTCTAGCATAT

human ----ACAGTATT-GTG-CAGCC------------ATCACCA--CCATC-------CATCT

rat AGCTGCAGTCCTCATGGCTTCCCAC-TTCTCTTAACTACGGAGCCGTCTCTCTAGCATAT

zebrafish AACAAAACTTTTCCAGAATAC------------AGCCACTTTGCTATT---------TTT

* * * * * ** * * * *

mouse CCAGTTTAATTTTTGG--ACCTTCTAGGTCATTGACTTCTCTTGGGCCACTGGTCCAACC

human CCAGA--AGTCTTC----ATCTTGCAAA--ACTGAACTCTGTCC--CCATTAG----ACT

rat CCTGTTTAATTTTTGT--GCTTTCTAGGCCACTGACTTCTCATGGGCCGCTGGCCCATCT

zebrafish TTAGG--AGGTGTTGAGAATGTTATATA-CAC-AACCCCTGTAAA-CTGGTGGGTGGATG

* * * ** * * * ** * * *

mouse GTGACCCA---CT--GTAGTGCTTTCATGT------CACGGGTAGTTAAGCTCCCAGCT-

human CTCACTCC---CC--ATT-TGCCCTC----------CCCTGGCACCATGGCTCACACCTG

rat GTGTCCCA---CTGTATAGTTCTTTCTTGTCATAGTCATGGGTAGGTGCGCTCACCACT-

zebrafish ATAACATGGGGCCTAGTTAAGCTTTGGTAAGG-----CCTGTTGTTATGGTT-ACATCTT

* * * * * * * * * * **

mouse TG-GTGCTCGGTAGT--GTGCAGGGTCTCTACTTGGAGGTCAGTGGTCTTTGGCTCCTTG

human TA-AT-CCCAGCACT--TCGGGAGGCT--------GAGG-CAGGAG-------------G

rat TA-GTGCTGTGCAGC--GTACAGCGCCTCTGCTTGGAGGCCACTGGTCTTAGGCTCCTTG

zebrafish TGCATGCTTTTCGGCTGGTAAAAAGCACC-----ATAAATGAGAAGG---------CTCG

* * * * * * * *

mouse ACTATTTTTCCTTCTGACCTGGAGTGTGTCAGCACTTCGGCGGACCTGTGCTCTGGCACA

human ACCA------CTCCAGCCCAGGAAT---TCGATACC------AGCCTG------GGCA--

rat TCTTTTTCT-CCTCTGACCTGGAGCGTGTCGGTACTTCAGCTGATCCGGGCTCTGGTACA

zebrafish ACCTC-----CTTTAG---TAGACT----CGG--CT------ATTCTG------GGTAC-

* * * ** * * * * ** *

mouse GATGTGGACATGTGTTGCGGGTGGAGGG----------GTGCTTGTGTTTGACCAGAGGA

human -------ACATA------------GGGA----------GACCCTGTCTCT-ATAAAAAAT

rat GATGTGGATGTGTGTTGGGGCGGGGGGGTGGTGGAAGTGTGCCTCTGTTTGACCAGAGGA

zebrafish --------TACC------------AGAA-----------GCCCTGGGTTT--TGAGATCT

* * * * * * *

mouse TGATGACTGCTTTGTTCTGCCTGGTGCCTCCAAGGACATGTGTGGTCCCTGTACTTTTCT

human TAAAAA--AATTAGCCGTGTGTGGTGGCAC-----GCACCTGTAGTCCCAGC----TTCT

rat AGATGGCTGCTTTGTTCTGCCTGGTGCCTCCACGGGCCTGTGTGGTAATTGTACTTTTCT

zebrafish TAATG-----TTCGCATGAAGAAATAACTT--------CCTATGA-ACCTGT----TTGT

* ** * * * * * * ** *

mouse CTTTCATTTGGAAGATGCCAAAATTCTCTGTCACTCACGGCTGGCCCAGGCCAAAGACCC

human CAGAAAGCTG--AGGTGGGAGGATTGTTTGT------------GCCCTGG----------

rat CTTTCATTTGGAAAATGCCAAAATTCTCTGTCACTCACAGCTGGCCCAGGCCAAAGAGAC

zebrafish ATTATAAT----ATTTTAGACTATACCTTTTAA--------------AAGCAA-------

* * * * ** * * *

mouse TTGGATTAATGGTGCT-ATGGGGTGGTATCTTTCCTTTGTAAGCCAAGCTACACCAGATT

human --AGTTTGAGGCTACA-GTGAGCTGTGAT--------TGT--GCCA--CTGCACT----- 0

rat TTGGATTAATGCTGCT-GTGG-----TATTTTTCCCTTGTATGCCAAGCT----------

zebrafish -CATCTTAA-GCTGCAAGTGA----CAAT--------TGTA-GTTACG-TGAACTG----

** * * * * ** ** *** * * *

mouse ACTGGGATCTGTGGCTACTGCTTAGGACCTCATGGGAAGCCCGAGGAGCCAGCAACAGGG

human -CCAG--CCTG-GGCAACAGAGTGAGACCCTGTC--------------TCAGAAAGAAAG

rat -TCTG--CTTAGGACATCAAGGGAAGCCCGAGTGG-------------CCAGCAACAGAA

zebrafish --TAG---TTA-ATCATCAGCGCAATAT-TCATGA--------------TTGTAACAGA-

* * * * * * ** *

mouse AAGCAGGTCTGCCTG-CTACTG----TCCTGAATGG---CTCCGACACTGAGCCTTGGTT

human ATCTTTGCCTGCCTGGCAACTGACTTTTGTGAATGA---C-CAGATACAGTGACTTA---

rat ---CAGGTCTACCTGCTACCTG----TCCTGACCGGAGGCTGAGACTCTGAGCCCTTG--

zebrafish -------CCTGCATG---ACAG----TTTTAAACAAGAATGCAGACTCTTTGCTATGT--

** * ** * * * * * ** * * *

mouse TTGTTTGCTTTTGGTTTGCTTTGTTTTGTTTGTTTTTGAGTCAGTG--TCTCTCATGTAA

human --------TTTCGAATTGCTGT-------------TTGAGT-GGTGGATCTCTGATGAA-

rat --------TTTTGTCTGCCTGT------TTTGTTTTGGAGTCAGTG--CCTCTTACATAA

zebrafish --------TAATGAGCTGTTGAG--------GATGCTATATACACA-ATCTCT-ATATAA

* * * * **** * *

mouse CC-CCGGCTGTCCTGGTACTCAGTATGTACACCAGGCTGGCCTCAGACTCAGAGATCCTC

human ---CTGGC---CCCTTCCCTC----------CC---CTAGCCTCAGGTTC-------CAC

rat CC-CTGGCTGTCCTGAAACTCAGTATGTAGACCAGGCTAGCCTCAGACTCAG--ATCCTC

zebrafish GAACTGGTGG--GTGGAAATGA---------------TAGCATGGGGCCTAGTTATGCTT

* ** * * ** * * *

mouse CTGCCTTTGCCTCTTGAGTGTTGGGATGGAAGGTGTGAGCCACCGCTCCAGGCTTGCCTC

human CTG-------------------GGGATG------------------TTTGTGTATTCTTT

rat CTGCCTTTGCCTCCTGAGTATTGGGAGAGAAGGTATGAGCCACCGCTCCAGGCTTGCCTC

zebrafish TGG-----------------TAGGACTGGTTG---------------TTATGGTTACATC

* ** * * * *

mouse TGTTTTTGAAGAGAAACACGTAAGAAGGACCTGTGTCTGTACGTTGATTGATTTCATAGG

human TGTAAATGATGAATAAT--GGAGGAAAAACC-GTATCAATA-ATT--TTCACCTT-TAAT

rat TGTTTTTGAAGAGAAACATGTATGAAGGACCCGTGTCTGCATGTTGACTAATTTCATGGT

zebrafish TTTGCATGCTTTTTGGCTGCTAAAAAGCGCC-------ACACATT-------CTTA-AAG

* * ** * ** ** * ** *

mouse GAGC---GACTAGGCTCAGGGATTTTGATT-TTGATTTTTTTTTTCCTCAAGAGACAGGG

human CAGC---ACCTCG-----------TTCACC-TTTGCCGTATTTTTATTAAATGTGCAGGT

rat GAGC---GACGAGGCCCGGGGATTTTTATT-TTGAATGATTATTTGC--AATATGAATCT

zebrafish GAGCCTTATTTAAATTCAGG---TTACATTGTCTCATGTCTCCTTTTTTAATA-GCTGTT

*** * * * * ** **

mouse TTTCTCTGTGTAGCCCTGGCTGTCCTAGAACTCACTTTGTAG--ACCAGGCTGGCCTCGA

human GCTCCTTG----GCCTTTGCTG------AGGTCACGCCTTTG--ACCA--CTGTCAGTGG

rat GCCCTTT------CATTT------------CTCATGTATCAGGGAATATATATACTTCAG

zebrafish TTTCCC------ACTCTTAATGGC-----GTTGAATCTTTCATGCTCAGACGCTCATTCT

* * * * * * *

mouse ACTCAGAAATCCGCCTGCCTCTGCCTCCCAAGTGCTGGGA--TTAAAGGTGT-GTGCCAC

human A----------------------------AGGCGTTGTGA--GTCGAGGAAC-GTGCCGA

rat G----------------------------AAGTAATAAAC--GATGAAGAACAGTGCTTT

zebrafish ATT--------------------------AAACTATGAGGCCATCGAAAGAT-TTACCGT

* * * * *

mouse CACCGCCCGGCAGATTTTT-ATTTTGA-ATTATTATTT-GCGATAGGATTCTGCCCTCTC

human -AGTGCCTG-------CTC-CTTTTGC-CCCATCACCAAGCTGAAAAGTCCTA-------

rat GACC-TTTA-------CTCACTCTCGCGCTCATCGCCA-GCCAT--GTTTTTA------- 6

zebrafish --TTTTTTA--------TC-TTTCAGCAAGCTTCATTT-GTTAT--GCCTCCA-------

* * * * *

mouse ATTTCCCGTGTATTGGGGACAGGTAGACCTTCAGATATACTTCAGCAAGTAAGAAATGAC

human ----------------AGTCAG--AGACTCTCAAATA--CT-----------GAAATTGT

rat ----------------------------TTTAATATAATTT----------AAAAATCGT

zebrafish --------------GATGTCA-----ACTTCCTTATTCGTT-------------AGTTGA

** * * *

mouse GAAGGAAGAATAGTACTCTGACCTTTGCTTCTCTCGTGGCCATTTTTCTATTTGACGTAA

human GTTGAAAG---------------------TCCCTTACAGCCATTTGCTT--TTAACCAAA

rat GACGGGAT---------------------TTCCCAGCAGTCACTTGTGT--TAGCCCAAG

zebrafish ATTGACAC--------------------CCTTCGGGTAAACGTTTACAC--TTTTCCAAC 2

* * * * ** * * *

mouse TTTAAAAATCGTGATGGAGTTTCCCAGCACTTACTTGCTTCAGCCACAGTTGTTG-TAAC

human TT-----GTTTTAACGAAA-----------------GCTTTAACCATTTTTCTCGATAGC

rat CT-----GTTTTGACAG------------------CGGTCTGCCCCTTTTTCTCAACAGG

zebrafish TT------GTGTGATGA----------------------CTGG-----TTTCCTG---TC

* * * **

mouse TGAGGTCTGCCACCTTTTCCCAACAGCTTCCTGCTGAACCGGGAGTTAGAAGAGTGCCGA

human TGAAAG--ACCAGGTTTTA---------TCCT--TAAGTCACGAGATAGAAGAGTGCCGC

rat TGAGGG--ACCAGGTCTT-----------CTTGCTGAGCCGGGAGTTAGAGGAGTGCCGA

zebrafish TGGGGT--GTCTCTCCTGCC--------TCTTCTTCAGCTGCGAG-CAGAGG--------

** * * * * * * *** *** *

mouse GCGGAGCTGGAGCAGCTACAGCAGAGGCGGGAACGGGAGAACCAGGAGGGCACCACGCTG 4

human TCCGAGTTGGAGGTGCTGCAGCAGAGGCGGGAGCGGGAGAACCGGGAAGGCGCAAACCTC

rat GGGGAGCTGGAGCAGCTGCAGCAGAGGCGCGAGCGGGAGAACCAGGAGGGCGCCACTCTG 9

zebrafish TCAG---TGGA----CTACAGG------GGGAGCTGGAGGGCAAAGGGG--------CTG 6

* **** ** *** * ** * **** * * * **

mouse ATCTGCATGCTCAGGGCTGACCTGGAGCTGGCTCAGGGCGAAGGG 6059

human CTCTCCATGCTCAAGGCCGAC------------------------ 4267

rat ATCTGCATG------------------------------------ 9178

zebrafish AA----ATG------------------------------------ 3951

***
